# Complete Genome Sequence of *Labrenzia* sp. Strain CP4, Isolated from a Self-Regenerating Biocathode Biofilm

**DOI:** 10.1128/genomeA.00354-16

**Published:** 2016-05-12

**Authors:** Zheng Wang, Brian J. Eddie, Anthony P. Malanoski, W. Judson Hervey, Baochuan Lin, Sarah M. Strycharz-Glaven

**Affiliations:** Center for Bio/Molecular Science and Engineering, Naval Research Laboratory, Washington, DC, USA

## Abstract

Here, we present the complete genome sequence of *Labrenzia* sp. strain CP4, isolated from an electricity-consuming marine biocathode biofilm. *Labrenzia* sp. strain CP4 consists of a circular 5.2 Mbp chromosome and an 88 Kbp plasmid.

## GENOME ANNOUNCEMENT

*Labrenzia* spp. are aerobic, motile, Gram-negative bacteria recently assigned to the family *Rhodobacterales* (1). Currently, the genus *Labrenzia* consists of five recognized species: *L. alexandrii*, *L. aggregata*, *L. alba*, *L. marina*, and *L. suaedae* (1, 2), all isolated from hypersaline habitats. However, the only complete genome currently available is that of *L. alexandrii* type strain (DFL-11^T^) (3). The general characteristics of this genus include polar flagella, ubiquinone 10 (Q10) as the major respiratory lipoquinone, nitrate reduction to nitrite or nitrogen, and chemoheterotrophic and nonfermentative growth under aerobic and anaerobic conditions (2). Notably, *Labrenzia* spp. have been isolated from microbial communities enriched from the Deepwater Horizon (DWH) site, as well as other oil-contaminated coastal marine sediments (4–6), and found to degrade polycyclic aromatic hydrocarbons (PAHs).

*Labrenzia* sp. strain CP4 was isolated from a biocathode microbial community, known as Biocathode-MCL (*Marinobacter*, *Chromatiaceae*, *Labrenzia*), originally enriched from seawater collected at Rutgers Marine Field Station (RUMFS) in Tuckerton, New Jersey, USA (7). Biocathode-MCL uses electrons supplied by a cathode to drive CO_2_ fixation and O_2_ reduction and *Labrenzia* has been shown to be one of the most abundant active constituents (8, 9). Genome analysis of CP4 supports ongoing multiomics studies of Biocathode-MCL to determine the mechanism of extracellular electron transfer (EET) at the biocathode for microbial electrosynthesis (8, 9).

The genome of CP4 was sequenced by DNA Link USA, Inc. (San Diego, CA, USA) using the PacBio RS II sequencing platform (Pacific Biosciences, Menlo Park, CA, USA). Genomic DNA was extracted using the Wizard genomic DNA purification kit (Promega, Madison, WI, USA) and used to prepare a 10-kb insert library that was sequenced using two single-molecule real-time (SMRT) sequencing cells and P4-C2 chemistry. This resulted in 17,249 filtered and preassembled sequence reads with a mean length of 7,314 bp and 111× genome coverage. Assembly (via SMRTpipe HGAP.2 and SMRTpipe Celera Assembler) and consensus polishing (SMRTpipe Quiver) yielded two circular contigs with sizes of 5,249,082 and 87,984 bp (58.97% G+C content), which represent a chromosome and a plasmid, respectively. The genome is predicted to contain 5,746 protein-coding sequences (CDS), 3 rRNA operons, and 60 tRNAs using RAST (Rapid Annotation using Subsystem Technology). *Labrenzia* spp. are known to oxidize CO to CO_2_ by proteins encoded by the carbon monoxide dehydrogenase (CODH) operon (*coxLMS*) (10, 11). Identification of two forms of CODH (form I and II) in CP4 suggests that this bacterium has this capability. It is noted that CP4 contains homologous genes to those for the photorespiratory glycerate pathway in cyanobacteria (12). However, in contrast to *L. alexandrii*, CP4 does not contain the photosynthetic reaction center genes *pufLM* or bacteriochlorophyll synthase genes. The plasmid shares many features with plasmid LADFL_5 in *L. alexandrii*, such as a P-type ATPase translocating heavy-metal ions and mercury and cobalt-zinc-cadmium resistance systems (3). In addition, it also harbors multiple genes encoding cytochrome *c* and cytochrome oxidase biogenesis proteins.

### Nucleotide sequence accession numbers.

The complete genome sequences of *Labrenzia* sp. CP4 have been deposited in GenBank under accession numbers CP011927 (chromosome) and CP011928 (plasmid).
